# First-Principles DFT Investigation of CsSn_0.5_Ge_0.5_I_3_ and Machine Learning-Assisted Numerical Simulation of Lead-Free Solar Cells

**DOI:** 10.3390/ma19153341

**Published:** 2026-08-06

**Authors:** Qinmiao Yu, Jinglan Liang, Xueji Chang, Xiaojuan Xia, Jiang Zhao

**Affiliations:** College of Integrated Circuit Science and Engineering, Nanjing University of Posts and Telecommunications, Nanjing 210023, China

**Keywords:** lead-free, perovskite solar cells, CsSn_0.5_Ge_0.5_I_3_, first-principles calculation, SCAPS-1D, machine learning

## Abstract

The optoelectronic properties of the lead-free perovskite CsSn_0.5_Ge_0.5_I_3_ are investigated by first-principles calculations and numerical simulations using SCAPS-1D. The energy-level alignment between transport layers and the perovskite layer is evaluated, resulting in the establishment of the PCBM/CsSn_0.5_Ge_0.5_I_3_/PEDOT:PSS structure. Key parameters, including bulk defect density, layer thickness, and electrode materials, are optimised, and the effects of resistance, illumination intensity, thermal stability, and carrier generation-recombination rates on device performance are analysed. The optimal device structure FTO/PCBM/CsSn_0.5_Ge_0.5_I_3_/PEDOT:PSS/C achieves a power conversion efficiency (PCE) of 24.50% and a fill factor (FF) of 80.01%. Machine learning (ML) algorithms are applied to predict photovoltaic parameters, with Random Forest (RF) exhibiting the highest accuracy. SHAP analysis identifies absorber layer thickness as the dominant factor influencing efficiency, providing guidance for experimental optimisation. This integrated approach offers a practical pathway for designing high-performance, stable, and environmentally sustainable perovskite solar cells (PSCs).

## 1. Introduction

The emergence of inorganic–organic halide perovskites is recognised as a breakthrough in the development of high-efficiency photovoltaic materials [1]. Over recent decades, solar cells based on these perovskites are reported to have broad application potential owing to the properties of the perovskite light-absorbing layer. These properties include simple fabrication, tunable band gap, high absorption coefficient, long carrier diffusion length, high charge-transport efficiency and extended carrier lifetime [2,3,4]. Advancements enabled by these attributes have increased the PCE of PSCs from the earliest reported value of 3.8% to values exceeding 26% [5,6]. Lead-based perovskites reach high efficiency, but the toxicity of lead and their instability in humid environments limit commercial viability [7,8]. This concern has led to extensive exploration of lead-free alternatives. Cations including Ca(II), Bi(III), Sn(II) and Ge(II) are identified as principal candidates for replacement [9,10,11,12]. These materials continue to face the challenge of instability under ambient conditions. In tin-based perovskites, the susceptibility of tin to oxidation from the +2 to the +4 state in air restricts device performance and operational lifetime [13]. Studies have reported that in tin-based PSCs with MASnI_3_ as the absorber, the PCE remains at about 6%, markedly lower than that of typical lead-halide perovskite systems [14].

A hybridisation strategy is employed in PSCs to enhance stability and improve overall performance. The approach involves systematic investigation of composition ratios in mixed-halide lead perovskites to simultaneously increase PCE and operational stability [15]. Thermal and light stability in lead bromo-iodide cells is reported to be improved through the incorporation of caesium (Cs) and formamidinium cations (FA) [16]. Band-gap tuning between 1.57 and 2.23 eV in (AzrH)PbBr_x_I_3−x_ mixed-halide perovskites is achieved by controlling the bromide to iodide ratio, providing a key advantage for tandem solar cell applications [17]. Mixed perovskites are consistently found to deliver higher efficiency and stability than single-halide analogues, and the development of improved mixed-halide compositions remains an active direction of research.

The all-inorganic lead-free perovskite CsSnI_3_ is considered a promising absorber material for PSCs owing to its near-ideal band gap of approximately 1.31 eV, strong light absorption, and high carrier mobility [18,19]. Thermal instability in CsSnI_3_ results in rapid degradation of device performance [20]. A Sn/Ge hybridisation strategy is adopted to construct the binary metal perovskite CsSn_0.5_Ge_0.5_I_3_, which is structurally stable and resistant to air exposure. PCE of up to 7.11% is reported for devices fabricated by alloying Ge(II) into CsSnI_3_ to form CsSn_0.5_Ge_0.5_I_3_ in combination with a native-oxide passivation process [21]. Substitution of part of the Sn with Ge is shown to enhance efficiency. In stoichiometric CsSn_0.5_Ge_0.5_I_3_, Sn I and Ge I anti-site defects are found to be benign, without introducing deep levels or serving as non-radiative recombination centres [22]. Favourable Goldschmidt tolerance and octahedral factors, calculated as 0.94 and 0.40, respectively, are identified as contributing to the stability of the crystal structure.

In recent years, exploration of high-performance perovskite solar-cell materials has been accelerated through the application of data-driven approaches such as ML [23]. ML is recognised as an important tool for the analysis and optimisation of complex material systems and is regarded as providing a systematic paradigm for the development and refinement of PSC technology [24,25]. Large-scale simulation data are efficiently processed using advanced algorithms, enabling systematic tuning of parameters across functional layers of the devices. Hidden physicochemical patterns and coupling relationships among parameters are revealed through this methodology, offering guidance for the enhancement of material performance and optimisation of device architecture [26].

First-principles calculations are conducted to systematically analyse the structural, electronic, and optical properties of the lead-free perovskite CsSn_0.5_Ge_0.5_I_3_. On this basis, the photovoltaic potential of the device structure fluorine-doped tin oxide (FTO)/ETL/CsSn_0.5_Ge_0.5_I_3_/HTL/Au is evaluated through simulations using the SCAPS-1D software. Energy-level alignment between the transport layers and the perovskite layer is identified as crucial for device performance, with appropriate band alignment shown to reduce interfacial recombination and facilitate carrier diffusion into the transport layers. Compatibility screening of 25 candidate electron and hole transport materials, based on efficiency and energy-level matching, results in the selection of [6,6]-phenyl-C_61_-butyric acid methyl ester (PCBM) as the electron transport layer (ETL) and poly(3,4-ethylenedioxythiophene):poly(styrene sulfonate) (PEDOT:PSS) as the hole transport layer (HTL). Further optimisation of parameters, including the doping concentration and thickness of the transport layers, absorber layer properties and metal back electrode, leads to marked enhancement in device performance. Non-ideal factors such as resistance, light intensity, and temperature are assessed, with results indicating significant application potential for CsSn_0.5_Ge_0.5_I_3_ as a lead-free absorber material. ML techniques are incorporated to extend the research methodology by predicting and interpreting photovoltaic performance metrics. The dataset obtained from SCAPS-1D simulations is used to develop supervised machine learning models for estimating key photovoltaic parameters. The evaluated algorithms comprised linear regression (LR), support vector regression (SVR), RF, and extreme gradient boosting (XGBoost, version 3.1.2). The integration of SCAPS-1D simulations with machine learning is found to provide a robust and generalisable design strategy, enabling accelerated development of efficient, stable, and environmentally sustainable lead-free PSCs.

## 2. Methodology

### 2.1. First-Principles DFT Study of CsSn_0.5_Ge_0.5_I_3_

Density functional theory (DFT) is a computational approach based on the electron density, through which the energies and related properties of many-electron systems are obtained by approximate solutions of the Kohn–Sham equations [27]. Owing to its high accuracy and reliability, DFT has become an indispensable tool for the investigation of material properties and has contributed to an improved understanding of the optical and electronic characteristics of materials from a first-principles perspective [28].

**Figure 1:**
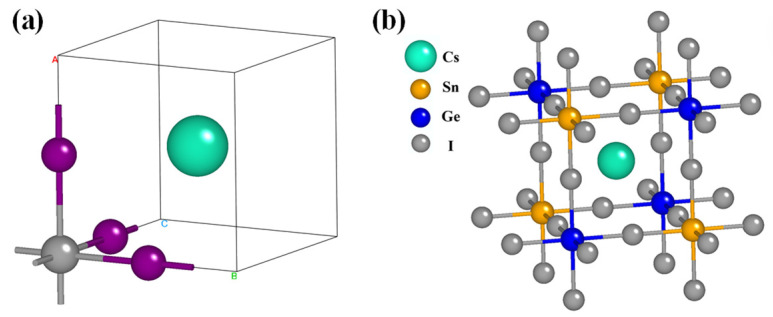
(**a**) Schematic illustration of the unit cell. (**b**) Crystal structure of CsSn_0.5_Ge_0.5_I_3_.

Device Studio [29] combines visualisation, modelling, and simulation capabilities within a unified platform, while first-principles calculations are performed through its integrated DS-PAW package [30]. First-principles calculations were conducted, with the electron–ion interactions described using the projector augmented-wave (PAW) method. The structural relaxation and convergence analyses were performed within DFT using the Perdew–Burke–Ernzerhof (PBE) exchange–correlation functional under the generalised gradient approximation (GGA) [31]. A plane-wave kinetic-energy cutoff of 500 eV was adopted. All atomic coordinates were relaxed through the conjugate-gradient method until the residual force on each atom was less than 0.01 eV Å^−1^. The self-consistent total energy was considered converged when the energy variation reached below 1 × 10^−6^ eV per atom. Brillouin-zone sampling was carried out using a 5 × 5 × 5 k-point grid. The unit cell used in the calculations is shown in Figure 1a and was described by the space group Pm3m. To model the mixed B-site perovskite composition AB′_0.5_B″_0.5_X_3_, in which Sn and Ge occupy the B sites in an equal 1:1 ratio, a 2 × 2 × 2 supercell was constructed. The supercell contains eight B sites, occupied by four Sn atoms and four Ge atoms. Sn and Ge were arranged in a fully ordered rock salt-type configuration, in which the two species alternate along all three crystallographic directions. Consequently, each Sn atom is surrounded by six Ge atoms as its nearest B-site neighbours, and vice versa. The resulting supercell contains eight formula units and has the integer composition Cs_8_Sn_4_Ge_4_I_24_, corresponding to the nominal composition CsSn_0.5_Ge_0.5_I_3_. The optimised geometric configuration is shown in Figure 1b. Since the HSE06 hybrid functional has higher accuracy than the traditional PBE functional in predicting semiconductor band gaps, both HSE06 and PBE functionals are employed for a comparative study in this work [32,33,34].

### 2.2. Numerical Study of SCAPS-1D

Theoretical analysis provides valuable guidance for experimental design. In the present work, the photovoltaic behaviour of the proposed device was evaluated using SCAPS-1D software (version 3312) [35]. The programme numerically solves the one-dimensional Poisson equation together with the carrier continuity and electron–hole transport equations to determine the current–voltage characteristics of the PSC. Further details were provided in the Supporting Information.

**Figure 2:**
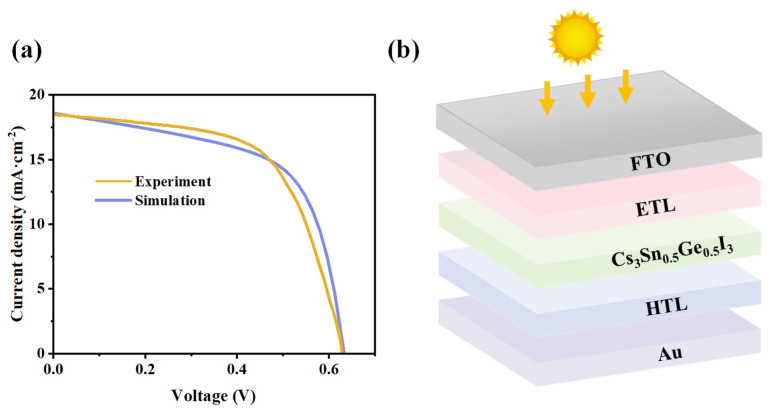
(**a**) Verification of the SCAPS-1D model. (**b**) Schematic of the device architecture.

To validate the initial parameter set, the FTO/PCBM/CsSn_0.5_Ge_0.5_I_3_/Spiro-MeOTAD/Au structure was reproduced using the parameters listed in Table 1 and Table S1, and the simulated results were compared with the experimental data. Here, Spiro-MeOTAD denotes 2,2′,7,7′-tetrakis[N,N-di(4-methoxyphenyl)amino]-9,9′-spirobifluorene. Considering potential experimental uncertainties, suitable series and parallel resistances were included in the simulations. The comparison between experimental and simulated results is presented in Figure 2a.

The device architecture investigated in this work, shown in Figure 2b, consisted of an FTO front electrode, a CsSn_0.5_Ge_0.5_I_3_ absorber, charge-transport layers, and an Au back contact with a work function of 5.1 eV. Input parameters for all layers and interfaces are summarised in Tables S2 and S3. Simulations were conducted under standard conditions at 300 K (unless temperature effects were explicitly examined) and under AM 1.5 G illumination (1000 W m^−2^).

### 2.3. Machine Learning

ML can identify underlying structures and correlations within complex datasets, enabling the development of predictive models and uncovering deep insights from existing or previously unseen information [39]. First, data preprocessing is performed, followed by partitioning the data into input features and target output parameters. On this basis, an ML model is constructed and systematically evaluated across three stages: training, validation, and testing. To obtain an unbiased assessment of model generalisability, the dataset was randomly divided into training and test subsets, with 20% reserved exclusively for testing. The remaining 80% was used for model development. Generalisation performance was evaluated by comparing the prediction results obtained for the training data with those for the unseen test data. The predictive accuracy was quantified using the root mean square error (*RMSE*) and coefficient of determination (*R*^2^), with their calculation formulas provided in Equations (1) and (2), respectively.

(1) $$RMSE=\sqrt{\frac{1}{n}\sum_{i=1}^{n}{\left(Y_{i}-{\hat{Y}}_{i}\right)}^{2}}$$

(2) $$R^{2}=1-\frac{\sum_{i=1}^{n}{\left(Y_{i}-{\hat{Y}}_{i}\right)}^{2}}{\sum_{i=1}^{n}{\left(Y_{i}-{\hat{Y}}_{i}\right)}^{2}}$$

Four machine-learning methods, namely LR, SVR, RF, and XGBoost, were investigated for predicting the target variable. Model development and evaluation were carried out in Python (version 3.12.4) using the scikit-learn (version 1.4.2) and XGBoost packages. Among these, the LR model captures linear relationships among variables and offers good interpretability, whereas SVR is a kernel-based statistical learning method capable of handling complex nonlinear relationships. To further enhance model performance and overcome the limitations of single models, ensemble learning techniques were introduced, with emphasis on the RF model, which is robust against overfitting, and the XGBoost model, which balances computational efficiency with predictive accuracy. Finally, SHapley Additive exPlanations (SHAP) analysis was performed on the best-performing model to quantify the importance of individual input features and visualise their contributions to the predicted results [40].

## 3. Results

### 3.1. Analysis of DFT Results

#### 3.1.1. Stability of CsSn_0.5_Ge_0.5_I_3_ Materials

In this study, all calculations were performed on the semiconductor material CsSn_0.5_Ge_0.5_I_3_ with the cubic Pm3m space group to achieve minimisation of its ground-state energy. The crystal structure is as follows: Cs atoms occupy the corner (1a) sites of the cubic unit cell with coordinates (0, 0, 0). Sn and Ge atoms are disordered and occupy the body-centred (1b) sites in a 1:1 ratio with coordinates (0.5, 0.5, 0.5), while I atoms occupy the face-centred (3c) sites with coordinates (0.5, 0.5, 0), (0.5, 0, 0.5), and (0, 0.5, 0.5).

The calculated lattice parameters are compared with the experimental data of CsGeI_3_ and CsSnI_3_, as presented in Table 2. The results indicate that the structure of CsSn_0.5_Ge_0.5_I_3_ lies between the ideal cubic phase and the distorted rhombohedral phase: its octahedral distortion factor (δ = 0.045) is significantly smaller than that of the rhombohedral phase of pure CsGeI_3_ (δ = 0.353) but larger than that of the cubic phase of pure CsSnI_3_ (δ ≈ 0). At the same time, its octahedral tilt angle also falls between the two pure phases. This clearly demonstrates that Sn alloying effectively suppresses the strong octahedral distortion in CsGeI_3_, driving the structure toward higher symmetry and thereby stabilising the perovskite phase [41]. This conclusion is fully consistent with the trends revealed by the tabulated data.

The structural stability of compounds in crystal structures is defined by the tolerance (t) and octahedral (*μ*) factors, given by the following formulas:

(3) $$t=\frac{r_{A}+r_{X}}{\sqrt{2}(r_{B}+r_{X})}$$

(4) $$\mu =\frac{r_{B}}{r_{A}}$$

Here, *r_A_*, *r_X_*, and *r_B_* correspond to the ionic radii of the cations occupying the *A* and *B* sites and the anion located at the *X* site, respectively. Coordination-dependent ionic radii are adopted, and their values, oxidation states, coordination numbers, and literature sources are summarised in Table S4. For the mixed CsSn_0.5_Ge_0.5_I_3_ composition, the effective B-site ionic radius is determined using the composition-weighted arithmetic mean. For an ideal cubic structure, the tolerance factor should lie between 0.9 and 1.0 [42]. In addition, the octahedral factor is another crucial parameter for assessing perovskite stability, defined as the radius ratio of the B-site cation to the X-site anion [43]. This parameter provides key insights into the stabilisation mechanisms of perovskite structures by characterising how well the B-site cation fits within the coordination space of the [X_6_] octahedron. It is widely accepted that a stable [BX_6_] octahedral configuration requires μ to be within the ideal range of 0.44 to 0.72 [44]. For CsSn_0.5_Ge_0.5_I_3_, its favourable tolerance factor (t = 0.938) and octahedral factor (μ = 0.398), as shown in Table 3, collectively contribute to the stability of this alloy structure.

**Table 2:** Perovskites compared with experimental data.

PSC	Structure	a (Å)	β (°)	δ	d_S_ (Å)	d_L_ (Å)	∠IMI’ (°)
CsSnI_3_	α ^a^	6.219	-	-	-	-	-
CsGeI_3_	α ^b^	6.05	-	-	-	-	-
	r ^c^	5.983	88.62	0.31	2.7526	3.2561	169.31
CsSn_0.5_Ge_0.5_I_3_	2 × 2 × 2	12.273	89.7	0.045	3.005	3.144	168.5

Calculated values of lattice parameters are compared to the results reported in the literature: ^a^, ref. [45]; ^b^, ref. [46]; ^c^, ref. [47]. α and r denote the cubic and rhombohedral phases, respectively, while 2 × 2 × 2 denotes the supercell used for CsSn_0.5_Ge_0.5_I_3_. The 2 × 2 × 2 supercell adopts a fully ordered rock salt-type Sn/Ge arrangement on the B sites and is compositionally equivalent to the ordered double-perovskite formula Cs_2_SnGeI_6_. β represents the rhombohedral interaxial angle, and δ denotes the off-centre displacement of the metal atom M from the geometric centre of the MI_6_ octahedron. d_S_ and d_L_ represent the shorter and longer M-I bond lengths, respectively.

**Table 3:** Tolerance factor and octahedral factor of CsSn_0.5_Ge_0.5_I_3_ materials.

	Ionic Radii (Å)	t	μ
r_A(Cs)_	1.88	0.938	0.398
r_B’(Sn)_	1.02		
r_B’’(Ge)_	0.73		
r_X(I)_	2.20		
experimental results [21]		0.94	0.4

#### 3.1.2. Electronic Properties

The electronic properties of materials reflect the motion and distribution patterns of electrons within their molecular or crystalline structures. Analysing the electronic band structure can aid in screening materials suitable for specific industrial applications [48]. To better understand the electronic behaviour of CsSn_0.5_Ge_0.5_I_3_, this study systematically analysed key electronic characteristics such as its bandgap and density of states.

**Figure 3:**
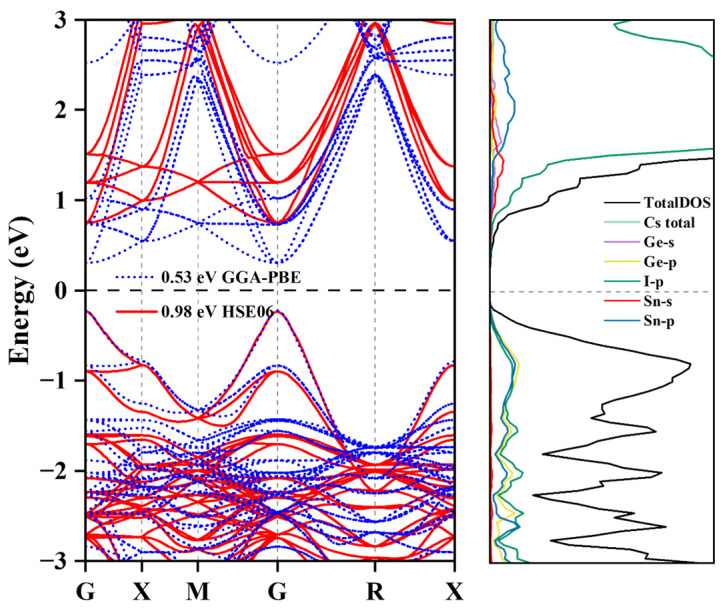
Bandgap structure and DOS of CsSn_0.5_Ge_0.5_I_3_ by GGA (blue dashed line) and HSE06 (red solid line) methods.

Table 4 summarises the electronic band-gap value obtained from the GGA-PBE calculations. The results obtained in this work are in good agreement with previously reported GGA-PBE values, indicating the reliability of the computational parameters and methods employed. However, the band gap calculated using the PBE functional is approximately 0.526 eV, which is significantly lower than the experimental value of 1.5 eV. This discrepancy mainly arises from the inherent underestimation of band gaps by semi-local functionals in describing exchange–correlation interactions. In general, this limitation can be addressed by employing hybrid functionals or the GW approximation. In particular, hybrid functionals improve the accuracy of band gap estimation by incorporating a portion of exact exchange. Previous studies have shown that such corrections primarily result in an upward shift of the conduction band, thereby enlarging the band gap, while the valence band structure and overall band dispersion remain largely unchanged [49,50]. To further validate the reliability of the present calculations, the band structure is recalculated using the HSE06 hybrid functional. The results show that the band gap increases to 0.98 eV, as shown in Figure 3, representing a significant improvement over the PBE value. Moreover, the overall band structure remains consistent with the PBE results. These findings further confirm the reliability of the computational approach in describing the electronic structure of the material. Nevertheless, the HSE06-calculated band gap remains lower than the experimentally reported value of 1.5 eV. Therefore, the experimental bandgap value is adopted as the absorber layer input in the SCAPS-1D simulations to represent experimentally relevant device conditions and to avoid propagating the residual DFT underestimation into the device-level calculations. The DFT-calculated band gaps are primarily used to analyse the intrinsic electronic structure and compare the performance of different exchange–correlation functionals, whereas the experimental value is used for macroscopic device simulation.

The electronic band structure of CsSn_0.5_Ge_0.5_I_3_ along the high-symmetry k-path G–X–M–G–R–X in the first Brillouin zone is shown in Figure 3. To accurately determine the bandgap, the Fermi level has been set as the reference zero point. Analysis indicates that both the valence band maximum (VBM) and conduction band minimum (CBM) of CsSn_0.5_Ge_0.5_I_3_ are located at the G point, confirming it as a direct bandgap semiconductor. Furthermore, the calculated partial density of states (PDOS) and total density of states (TDOS) for CsSn_0.5_Ge_0.5_I_3_ are presented in Figure 3. PDOS analysis reveals that the VBM of CsSn_0.5_Ge_0.5_I_3_ is primarily composed of Ge 4s, Sn 5s, and I 5p orbitals, exhibiting significant delocalised anti-bonding characteristics. The CBM is mainly formed by Ge 4p, Sn 5p, and I 5p orbitals. The synergistic interaction of these anti-bonding states not only shifts the conduction band to higher energy levels but also imparts favourable dispersion to the valence band. This unique band-edge structure facilitates a reduced effective mass of charge carriers and endows the material with potential for bipolar high mobility, thereby elucidating its excellent optoelectronic performance from an electronic-structure perspective.

#### 3.1.3. Optical Properties

Perovskite materials, owing to their unique and tunable optoelectronic properties, exhibit excellent optical responses upon interaction with light, demonstrating significant potential for a variety of optoelectronic device applications [52,53]. In this study, the key optical properties of the material, including reflectance, absorption coefficient, extinction coefficient, and refractive index, were systematically analysed to assess its potential for application in high-efficiency solar cells and other optoelectronic devices.

**Figure 4:**
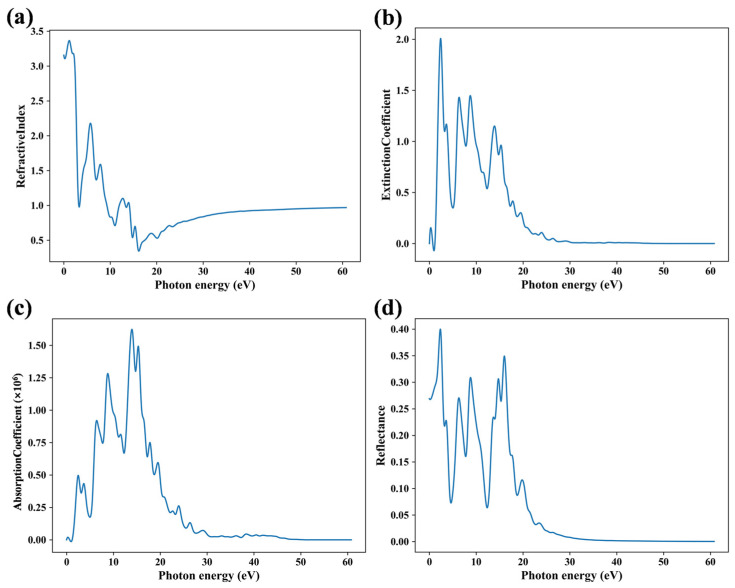
Optical properties of the material across the spectral range. (**a**) Refractive index (n). (**b**) Extinction coefficient (k). (**c**) Absorption coefficient α(ω). (**d**) Reflectance (R).

The refractive index is a complex number that describes the degree to which the propagation speed of light is reduced in a medium. Its real part determines the phase velocity, while its imaginary part (i.e., the extinction coefficient) characterises the attenuation of light. Their relationship can be expressed as Equation (5) [54]:

(5) $$\varepsilon (\omega )=\varepsilon_{1}(\omega )+i\varepsilon_{2}(\omega )$$

where *ω* is the frequency and $\varepsilon_{1}(\omega )$ and $\varepsilon_{2}(\omega )$ denote the real and imaginary parts of the dielectric function, respectively. Figure 4a,b displays the refractive index (n) and extinction coefficient (k) spectra of CsSn_0.5_Ge_0.5_I_3_. In the low-energy region (<1.3 eV), the k value approaches zero while the n value remains around 3.3, exhibiting typical normal dispersion behaviour. In the visible-light range (1.6–3.1 eV), the k value consistently stays at a relatively high level (>0.5), indicating strong light absorption within this spectral region. Meanwhile, the n value gradually decreases with increasing energy and shows a gentle variation trend. In the higher energy range (>10 eV), several broad peaks appear in both the n and k spectra, corresponding to electronic transitions from deep valence bands (such as I 5s, Ge 4s, etc.) to higher-energy levels of the conduction band.

The absorption coefficient α(ω) quantifies the ability of a material to absorb incident light by describing the attenuation of light intensity per unit propagation length. Its dependence on photon energy is illustrated in Figure 4c. As can be seen from the figure, in the energy range from infrared to visible light (1.0–3.5 eV), the α(ω) spectrum is smooth and lacks sharp characteristic peaks, displaying broad-band continuous absorption. This indicates that CsSn_0.5_Ge_0.5_I_3_ possesses efficient, wide-spectrum photon capture capability, effectively absorbing both visible and infrared radiation. Such optical properties suggest promising application potential in fields such as solar photovoltaics.

Reflectance represents the fraction of incident optical power returned from a material surface. It is an important indicator for assessing surface-related optical behaviour and determining whether an antireflection coating is required. Figure 4d presents the reflectance spectrum of CsSn_0.5_Ge_0.5_I_3_ over the energy range of 0–60 eV. At the low-frequency limit, the reflectance R(0) is 0.27, indicating a moderate dielectric screening capability of the material. As photon energy increases into the visible region, the reflectance shows an upward trend, which aligns with the material entering its intrinsic strong absorption region (corresponding to significant extinction coefficient k values) within this range. When photon energy further rises above approximately 30 eV, the reflectance rapidly decreases and approaches zero. Research indicates that CsSn_0.5_Ge_0.5_I_3_ material exhibits excellent optical response characteristics within the visible light spectrum, laying a solid physical foundation for its application as a high-efficiency lead-free perovskite photovoltaic absorption layer.

### 3.2. Analysis of SCAPS-1D Results

#### 3.2.1. Choice of ETL and HTL

The selection of suitable charge-transport materials is examined from several perspectives. Electrons and holes photogenerated in the absorber are extracted through the corresponding transport layers and subsequently collected by their respective electrodes. Consequently, proper energy-level alignment at the interfaces between the absorber and the charge-transport layers is essential for efficient carrier extraction and overall device performance [55].

The ETL plays an important role in determining the PCE of PSCs by facilitating electron transport and extraction. Appropriate energy-level matching between the ETL and absorber promotes electron transfer while hindering hole migration, thereby reducing interfacial carrier recombination. The conduction-band offset (CBO) is commonly used to characterise this interfacial band alignment and is calculated according to Equation (6) [56].

(6) $$CBO=\chi_{ETL}-\chi_{absorber}$$

where $\chi_{absorber}$ and $\chi_{ETL}$ denote the electron affinities of the absorber and ETL, respectively. This study selects different ETL materials (TiO_2_, PCBM, ZnO, IGZO, WO_3_) to investigate the influence of ETL energy levels on PSC performance. The results of the CBO calculations are provided in Table 5. For the ETL, when CBO < 0, a cliff-like structure forms at the ETL/absorber interface. The electron flow from the perovskite to the ETL is a downhill process, resulting in high interfacial transport efficiency. However, an excessively large negative offset can lead to energy loss. When CBO = 0, the band alignment between the ETL and the absorber is perfectly continuous, creating no barrier for electron injection and thus achieving optimal level matching. Therefore, the minimal cliff structure formed when PCBM has a CBO of 0 eV provides the best energy level alignment with the absorber CsSn_0.5_Ge_0.5_I_3_.

Complementary to the ETL, the HTL selectively extracts and transports photogenerated holes while restricting electron transfer. Favourable energy-level matching at the HTL/perovskite interface facilitates hole collection by the corresponding metal electrode and suppresses interfacial recombination, thereby improving the photovoltaic characteristics of the device. The valence-band offset (VBO) is used to quantify the band alignment at this interface and is determined using Equation (7) [56].

(7) $$VBO=(\chi_{HTL}+Eg_{HTL})-(\chi_{absorber}+Eg_{absorber})$$

where $Eg_{absorber}$ and $Eg_{HTL}$ are the band gaps of the absorber and HTL, respectively. Several HTL materials, including CuSCN, poly(3-hexylthiophene-2,5-diyl) (P3HT), PEDOT:PSS, Spiro-MeOTAD, and CuI, are selected to investigate the effects of HTL energy-level alignment on photovoltaic device performance. The calculated VBO values are presented in the table. For the HTL, when VBO < 0, a cliff-like structure forms at the HTL/absorber interface. The hole transfer from the perovskite to the HTL becomes a downhill process with a strong driving force, resulting in high interfacial transport efficiency. As shown in Table 6, the device employing PEDOT:PSS as the HTL demonstrates the best performance. This can be attributed to the minimal cliff structure formed by the CsSn_0.5_Ge_0.5_I_3_/PEDOT:PSS alignment, which effectively reduces interfacial carrier recombination [57].

**Figure 5:**
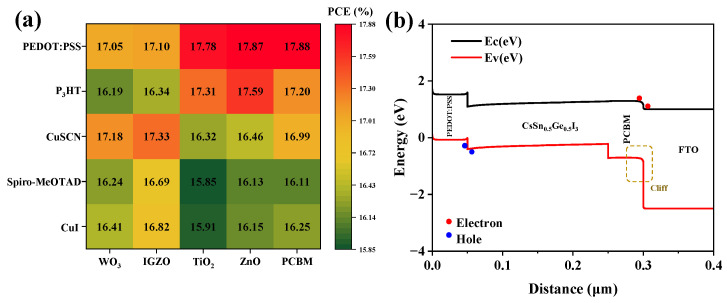
Influence of transport layers and material band structure. (**a**) Effect of different ETLs and HTLs on the performance of CsSn_0.5_Ge_0.5_I_3_ PSCs. (**b**) Representative band structure of the constituent materials.

An investigation is conducted on the PCE distribution of 25 CsSn_0.5_Ge_0.5_I_3_-based PSCs under the condition of fixed ETL and HTL thicknesses (0.05 μm); the results are shown in Figure 5a. The figure demonstrates that, regardless of the order of discussion, PEDOT:SS and PCBM are consistently identified as the optimal HTL and ETL for CsSn_0.5_Ge_0.5_I_3_, respectively, yielding a champion PCE of 17.88%. Concurrently, it is observed that the ETL exerts a greater influence on the PCE than the HTL. A plausible explanation for this is the inherent difference in electron and hole mobility within the perovskite, which makes interfacial recombination at the ETL side a more critical loss mechanism, thereby affecting the overall device performance more significantly [58]. Figure 5b shows the band alignment corresponding to the FTO/PCBM/CsSn_0.5_Ge_0.5_I_3_/PEDOT:PSS/Au device structure. At the PCBM/CsSn_0.5_Ge_0.5_I_3_ interface, an ideal band alignment is established through a flat CBO and a cliff-like VBO. This band alignment allows electrons to flow smoothly from the absorber to the ETL while blocking holes. Meanwhile, at the CsSn_0.5_Ge_0.5_I_3_/PEDOT:PSS interface, the band alignment forms a moderate cliff structure, effectively suppressing interfacial non-radiative recombination and enhancing device performance.

In the following discussion, the FTO/PCBM/CsSn_0.5_Ge_0.5_I_3_/PEDOT:PSS/Au structure is adopted as the standard architecture of PSCs for simulation and optimisation.

#### 3.2.2. Impact of Transport Layer Thickness on PSCs

**Figure 6:**
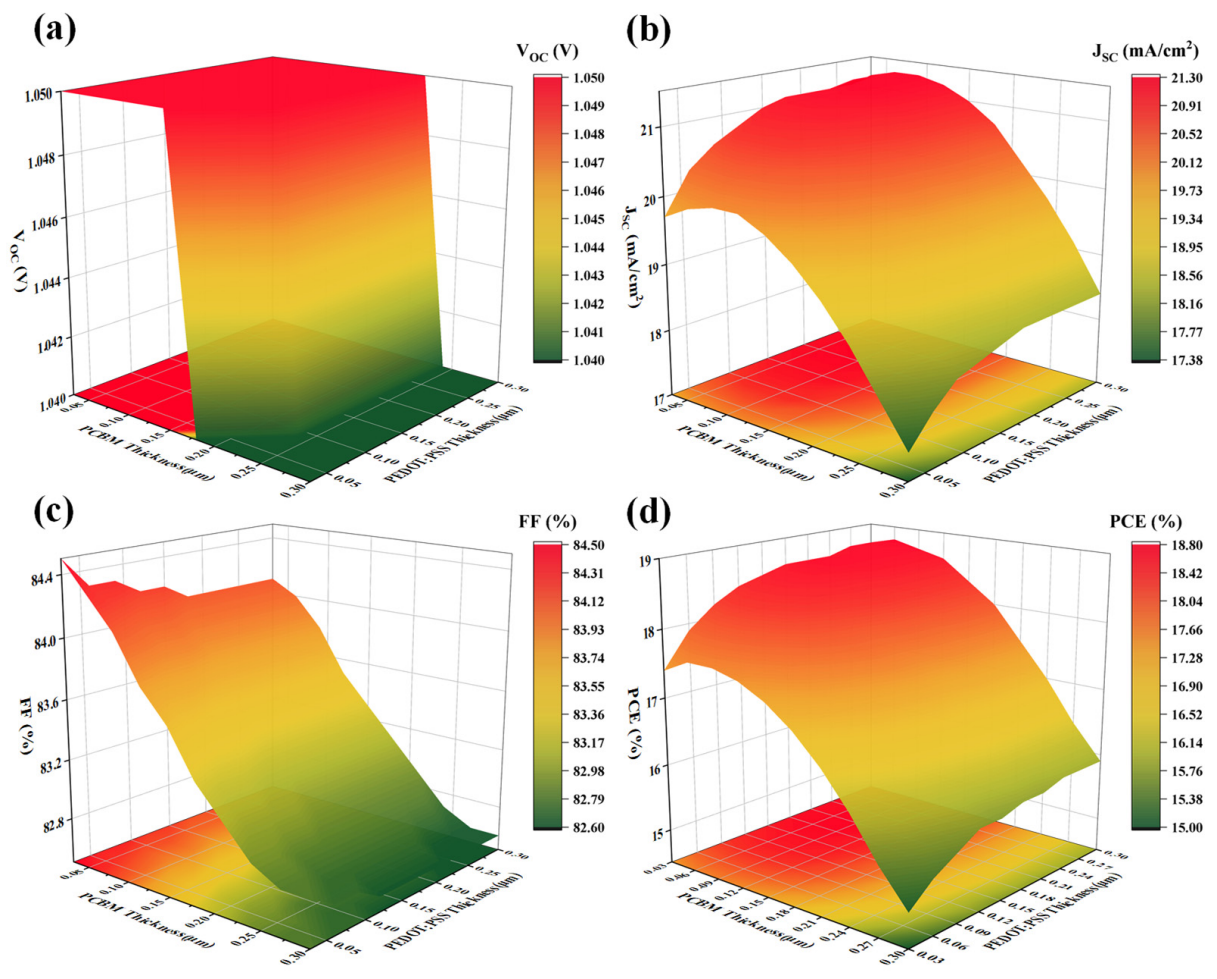
Effect of transport layer thickness on PSC performance. (**a**) V_oc_. (**b**) J_sc_. (**c**) FF. (**d**) PCE.

The optimisation of HTL and ETL thickness is a critical process to balance charge extraction efficiency and series resistance (R_s_). To investigate the optimal thickness of the charge transport layers, this study systematically varied their thickness (0.03–0.3 μm) and analysed the impact on device performance. The results are shown in Figure 6.

As illustrated in Figure 6, increasing the ETL thickness leads to a progressive deterioration in the overall photovoltaic characteristics of the device, whereas changes in the HTL thickness produce only a limited effect. This asymmetry reflects the inherent differences in transport dynamics between electrons and holes [59]. When the PEDOT:PSS and PCBM thicknesses are 0.3 μm and 0.06 μm, respectively, the device achieves the highest PCE of 18.76%. This phenomenon can be attributed to the following mechanisms: a thinner ETL helps minimise the R_s_ along the electron transport path, fully leveraging the advantage of high electron mobility, while a thicker HTL facilitates the formation of a dense, pinhole-free film, effectively blocking electrons and reducing interfacial recombination, thereby ensuring efficient collection of holes with relatively lower mobility.

#### 3.2.3. Impact of Absorber Layer on PSCs

As the primary photoactive component of a solar cell, the absorber layer governs photovoltaic conversion through light absorption, charge-carrier generation and separation, carrier transport, and interfacial charge collection. Because the optical response, defect density, and recombination behaviour of the perovskite absorber strongly affect device characteristics, the combined effects of absorber thickness and defect density on PSC performance were systematically investigated.

Defect density fundamentally constrains the theoretical upper limit of open-circuit voltage (V_OC_) and carrier diffusion length by governing the intensity of non-radiative recombination, while the thickness of the absorber layer directly modulates the competitive balance between light absorption efficiency and charge collection probability.

**Figure 7:**
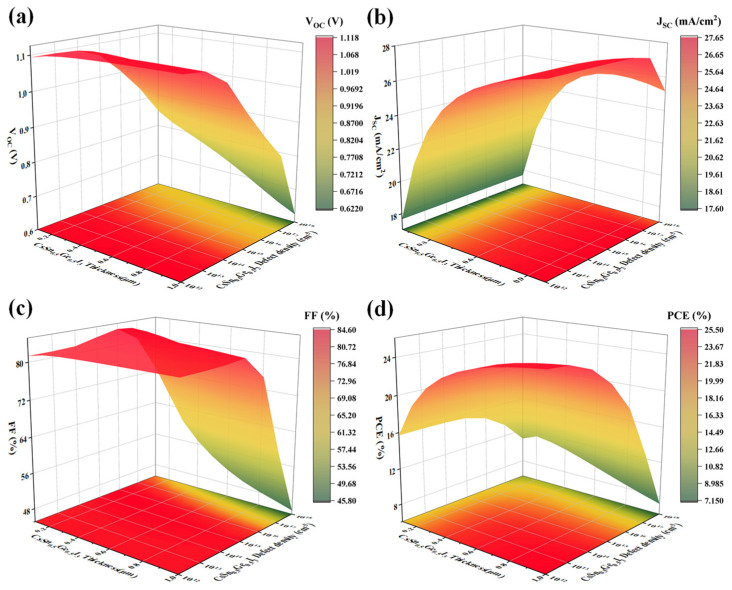
Effect of CsSn_0.5_Ge_0.5_I_3_ layer thickness and defect density on PSC performance. (**a**) V_oc_. (**b**) J_sc_. (**c**) FF. (**d**) PCE.

The CsSn_0.5_Ge_0.5_I_3_ absorber thickness varied between 0.1 and 1.0 μm, while its defect density was adjusted from 10^12^ to 10^18^ cm^−3^. Figure 7 presents the resulting changes in the photovoltaic parameters as functions of these two variables. The results indicate that superior device performance is achieved with a thicker absorber and a lower defect concentration. In particular, the short-circuit current density (J_SC_) rises markedly as the absorber becomes thicker. This trend is mainly attributed to enhanced photon absorption and the consequent generation of more photogenerated charge carriers, leading to improved light-harvesting capability.

After selecting an absorber layer thickness of 1 μm, the influence of defect density is further investigated. The defect density in the PSC is described using the Shockley–Read–Hall (SRH) model to characterise the non-radiative recombination process occurring via defect energy levels. The collective properties of these defects, such as density, energy level, and capture cross-section, together constitute the recombination model of the device [60]:

(8) $$R_{SRH}=\frac{p\cdot n-n_{i}^{2}}{\tau_{p}(n+n_{i})+\tau_{n}(p+n_{i})}$$

where *p* and *n* denote the hole and electron concentrations, respectively, $\tau_{p,n}$ and $\sigma_{p,n}$ are the lifetimes and trapping cross sections of the carriers, respectively. To more intuitively quantify the severity of such composite losses, carrier lifetime and carrier diffusion length are introduced:

(9) $$\tau_{p,n}=\frac{1}{\sigma_{p,n}\cdot \nu_{th}\cdot N_{t}}$$

(10) $$L_{n,p}=\sqrt{(\frac{\mu_{n,p}kT}{q})\tau_{n,p}}$$

**Figure 8:**
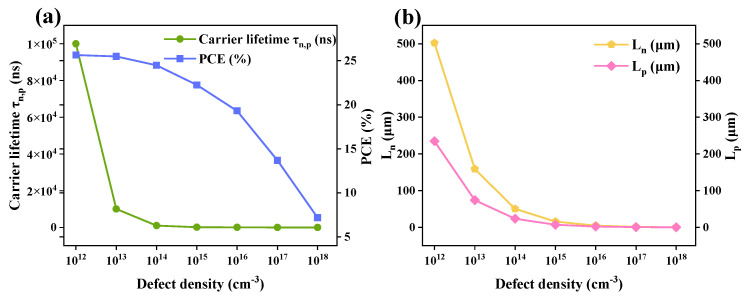
Effect of N_t_ in CsSn_0.5_Ge_0.5_I_3_ on optoelectronic properties and device performance. (**a**) Carrier lifetime ($\tau_{p,n}$) and PCE; (**b**) carrier diffusion length (L_n_ and L_p_).

Here, $V_{th}$ denotes the thermal velocity, which was set to 10^7^ cm/s based on the literature [61], while $\mu_{p,n}$ represents the carrier mobility. When the thermal velocity and carrier capture cross-section remain constant, the carrier lifetime is inversely proportional to the defect density, N_t_. Therefore, studying the impact of defect density on device performance essentially involves investigating its regulation of this key physical quantity—carrier lifetime. As shown in Figure 8a, the PCE of the device exhibits a declining trend with the decay of carrier lifetime. With increasing defect density, the PCE decreases significantly from 25.64% to 7.18%. When the carrier diffusion length is less than the thickness of the absorption layer, the recombination of photogenerated excitons occurs, fundamentally limiting device performance [62]. As shown in Figure 8b, as the defect density increases from 10^12^ cm^−3^ to10^18^ cm^−3^, the electron diffusion length L_n_ drops sharply from 100 μm to 0.5 μm, while the hole diffusion length L_p_ decreases from 200 μm to 0.23 μm, which is less than the thickness of the absorber.

In summary, reducing defect density is theoretically crucial for improving device performance; however, due to limitations in current manufacturing processes, excessively low defect density targets are often challenging to achieve. Therefore, the defect density of CsSn_0.5_Ge_0.5_I_3_ is set at 10^13^ cm^−3^.

#### 3.2.4. Impact of the Back Electrode on PSCs

PSC performance depends strongly on the work function of the back-contact material. A well-matched energy level alignment between the back electrode and the HTL enhances the built-in electric field, promoting carrier separation and transport. Conversely, a mismatch can lead to contact barriers, increase R_s_, hinder carrier extraction, and consequently reduce the FF and short-circuit current.

**Figure 9:**
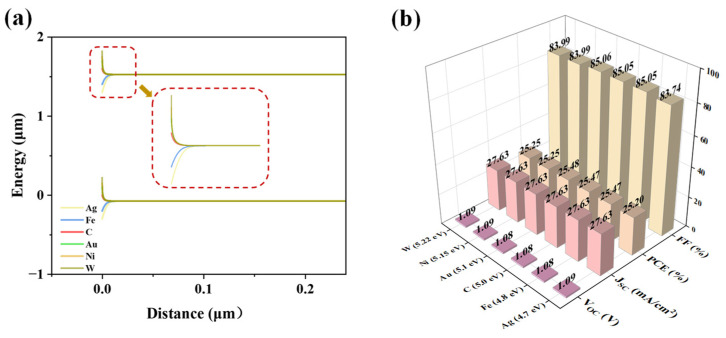
Effect of varying metal back electrodes. (**a**) Energy band structure. (**b**) Device performance parameters.

Ag, Fe, C, Au, Ni, and W are considered as back-contact materials for the PSCs, with corresponding work functions of 4.70, 4.80, 5.00, 5.10, 5.15, and 5.22 eV, respectively [63,64,65]. From Figure 9a, it can be observed that when the back electrode is Ag or Fe, a Schottky contact is formed at the HTL/metal interface, whereas when the back electrode is C, Au, Ni, or W, an ohmic contact is established. As the work function of the electrode increases, the nature of the interfacial contact gradually transitions from Schottky to ohmic. Meanwhile, as shown in Figure 9b, the J_SC_ and V_OC_ of the device remain largely stable, while the FF decreases significantly. This phenomenon can be explained by the fact that when the work function of the back electrode exceeds the optimal value for energy-level alignment with the HTL, the interface transitions from ohmic to rectifying contact, leading to an increase in contact resistance and consequently a reduction in FF. Considering both economic feasibility and material stability, C is chosen as the back contact material. The resulting device exhibited a PCE of 25.47% and an FF of 85.05%.

#### 3.2.5. Impact of Resistance on PSCs

The performance of solar cells is significantly influenced by the *R*_s_ and shunt resistance (*R_sh_*). Ideally, the device should exhibit zero *R*_s_ and infinite *R_sh_*. However, these effects cannot be completely eliminated in practical applications.

**Figure 10:**
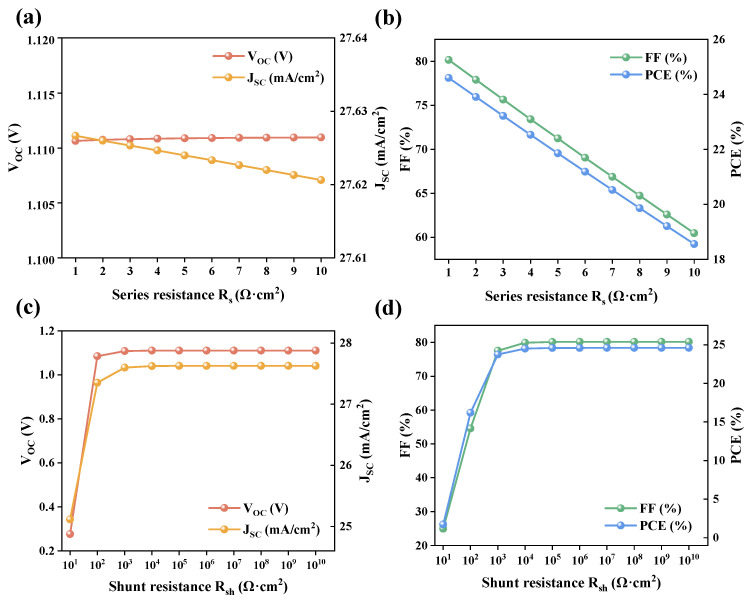
Effect of R_s_ and R_sh_ on the performance parameters of PSCs. (**a**) R_s_: V_oc_ and J_sc_. (**b**)R_s_: FF and PCE. (**c**) R_sh_: V_oc_ and J_sc_. (**d**) R_sh_: FF and PCE.

The *R*_s_ represents the combined resistive contributions from the absorber and interfacial layers, electrodes, electrical contacts, and interconnections within the device [66]. To assess how *R*_s_ influences the PCE, while maintaining an *R_sh_* of 10^5^ Ω·cm^2^, the *R*_s_ is gradually increased from 0 to 10 Ω·cm^2^, as shown in Figure 10a,b. With the increase in *R*_s_, the FF decreased significantly from 80.16% to 60.47%, and the PCE correspondingly dropped from 24.60% to 18.56%. *J_SC_* decreases slightly, while *V_OC_* shows negligible variation. The impact of R_s_ on solar cell characteristics is described by Equation (11) [67]:

(11) $$I_{SC}=I_{L}-I_{0}[\frac{qV_{OC}}{e^{nKT}}-1]-\frac{V_{OC}+I_{SC}R_{s}}{R_{sh}}$$

Here, $I_{L}$ denotes the photogenerated current, $I_{0}$ is the reverse saturation current, and n represents the diode ideality factor. The results demonstrate that increasing the *R*_s_ reduces *J_SC_* while causing more pronounced deterioration in the FF and PCE [68]. Accordingly, maintaining a low *R*_s_ is essential for limiting resistive power losses and improving the photovoltaic performance of the device.

In contrast, *R_sh_* is mainly determined by parasitic leakage pathways associated with bulk defects in the active layer, grain boundaries, and interfaces between adjacent layers [69]. To investigate the impact of *R_sh_* on device efficiency, the *R_sh_* is adjusted within the range of 10 to 10^15^ Ω·cm^2^ while maintaining an *R*_s_ of 1 Ω·cm^2^. The results are shown in Figure 10c,d. When the *R_sh_* decreases to 10 Ω·cm^2^, the PCE approaches zero due to severe shunting of photogenerated carriers caused by excessively low *R_sh_*, which significantly limits device performance [70]. As the *R_sh_* increases, key parameters such as *V_OC_*, *J_SC_*, FF, and PCE all show continuous improvement. Once the *R_sh_* exceeds 10^4^ Ω·cm^2^, device performance stabilises and remains essentially unaffected by further increases.

Seeking the optimal balance among cost, performance, stability, and manufacturability requires controlling resistance within the practical ranges mentioned above as a critical step. Based on this, when R_s_ and R_sh_ are optimised and fixed at 1 Ω·cm^2^ and 10^5^ Ω·cm^2^, respectively, the device ultimately achieves a PCE of 24.60% and an FF of 80.16%.

#### 3.2.6. Impact of Light Intensity on PSCs

Incident light intensity is an important operating parameter for PCEs. Under practical conditions, solar irradiance varies with location, season, weather, and time of day. Examining the photovoltaic response over a range of illumination levels, therefore, provides insight into device performance and operational robustness under variable outdoor conditions. The influence of light intensity on device performance essentially arises from its ability to alter the dynamic equilibrium of carrier generation, recombination, transport, and collection, which profoundly affects the interfacial physicochemical states of the various material layers. As the light intensity increases, the concentration of photogenerated carriers in the absorption layer rises significantly, leading to an enhanced splitting of the quasi-Fermi levels for electrons and holes, thereby causing the V_OC_ to increase logarithmically. However, the large current generated under high light intensity induces significant ohmic losses across the R_s_ [71]. Since the voltage loss increases quadratically with current, this results in a decrease in the FF [69].

**Figure 11:**
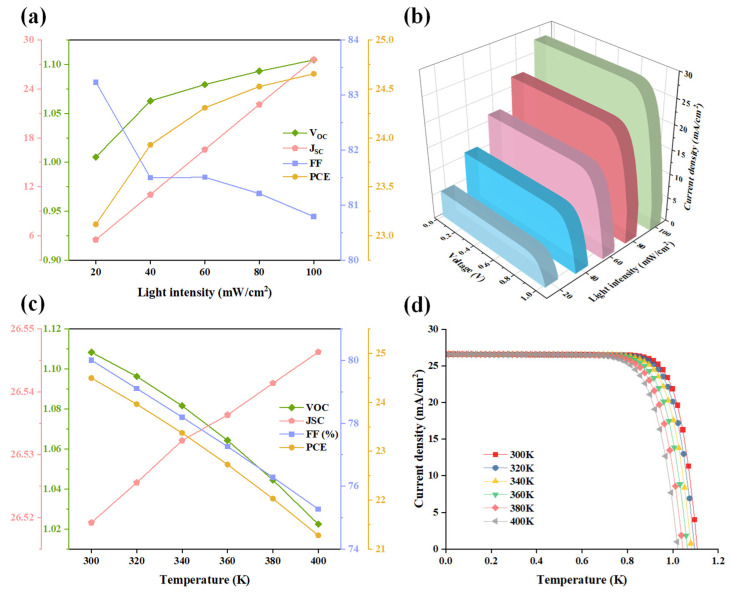
Effect of light intensity and operating temperature on the performance of PSCs. (**a**) Device performance parameters under varying light intensities. (**b**) Corresponding *J-V* curves under varying light intensities. (**c**) Device performance parameters under different operating temperatures. (**d**) Corresponding *J-V* curves under different operating temperatures.

Figure 11a,b present the variations in the photovoltaic parameters and JV characteristics, respectively, over an illumination-intensity range of 20–100 mW·cm^−2^. The variation in PCE reflects the dynamic balance between the gain from the enhancement of V_OC_ and J_SC_ and the loss in FF caused by intensified recombination and increased ohmic losses at high light intensities. As light intensity gradually increases, the overall shape of the *J-V* curve also shows systematic optimisation. At the same time, the impact of light intensity on PCE is less than 1.6%, demonstrating the device’s exceptional light adaptability. Since the nature of FTO reflection resembles a light-intensity mechanism, setting a 4% FTO reflectance better approximates real-world conditions [56]. This configuration yielded a PCE of 24.64% together with an FF of 80.88%.

#### 3.2.7. Impact of Temperature on PSCs

Temperature is a key factor influencing the stability of PSCs, and the ability to maintain high efficiency and stable operation across different temperature environments represents a core challenge for their commercialisation. To evaluate the influence of operating temperature on device performance and assess its thermal stability, Figure 11c,d show the temperature-dependent evolution of PSC performance parameters within the range of 300 to 400 K. As the temperature increases, the optoelectronic performance of the PSC remains robust, indicating that the device maintains excellent performance even under high-temperature conditions.

As the temperature increases, the band gap of perovskite materials contracts, leading to a reduction in the device’s V_OC_ [55]. Simultaneously, the anisotropy of mobility intensifies under high temperatures, hindering the effective transport of carriers. Furthermore, thermally induced interfacial defect recombination is significantly enhanced, disrupting the synchronisation between the carrier concentration gradient and charge extraction. This effect directly manifests as a decrease in the FF [72]. Collectively, these factors contribute to an accelerated carrier recombination rate, thereby reducing the overall PCE of the solar cell [73]. Overall, the PCE changes by less than 4% as the operating temperature increases from 300 to 400 K, indicating that the device maintains relatively stable photovoltaic performance over the investigated temperature range.

#### 3.2.8. Impact of the Generation and Recombination Rate

**Figure 12:**
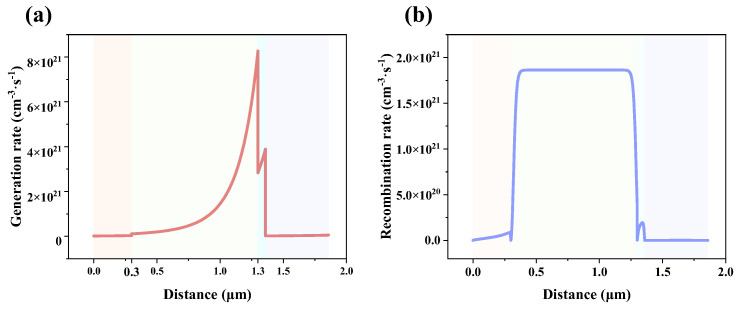
Effect of carrier generation and recombination rates on the performance of PSCs. (**a**) Performance vs. carrier generation rate. (**b**) Performance vs. carrier recombination rate.

Figure 12 shows the spatial distribution of carrier generation rate and total recombination rate in the optimised device. When the energy of incident photons equals or exceeds the band gap of the absorption layer material, electrons in the valence band are excited and transition to the conduction band, simultaneously leaving holes in the valence band, thereby forming electron-hole pairs. This photogenerated carrier generation mechanism is universally present in all pn junction photovoltaic devices. SCAPS-1D calculates the photogenerated carrier generation rate G(x) based on the photon flux $N_{phot}(\lambda ,x)$, determining the G(x) value for each wavelength range and position using Equation (12) [37].

(12) $$G(\lambda ,x)=\alpha (\lambda ,x)\cdot N_{phot}(\lambda ,x)$$

The generation rate profile shows a primary peak near the absorber/ETL interface, which is primarily attributed to the extremely high absorption coefficient of perovskite materials and the maximum electric field strength at this location. These factors significantly enhance the probability of photon absorption, leading to the highest photogenerated carrier generation rate. Meanwhile, a secondary peak observed near the ETL/FTO interface mainly stems from the reflection of incident light at this interface, causing localised enhancement of the optical field and thus forming a minor generation rate peak.

At the same time, the recombination rate peak appears in the bulk region of the absorber, indicating that poor crystallinity or high bulk defect density of the perovskite is the primary factor limiting device performance. Such bulk recombination directly reduces the V_OC_ and, due to its sensitivity to bias, further deteriorates the FF. Targeted bulk defect passivation (e.g., through additive engineering or crystallisation optimisation) is expected to significantly enhance device efficiency. Additionally, electrons in the conduction band and holes in the valence band recombine at a specific rate, which macroscopically balances the carrier generation rate. In PSCs, the recombination rate is jointly determined by carrier density and carrier lifetime.

#### 3.2.9. Predicting PCEs of Different Chosen PSCs with Supervised ML Models

Based on the defined range of absorber layer properties, device performance parameters were simulated and calculated using SCAPS-1D software, generating a dataset of 875 samples (Table S5). This dataset is further divided into a training set (700 samples) and a test set (175 samples), used for model construction and validation, respectively. The predictive capabilities of the four supervised machine-learning models are compared, and the resulting evaluation metrics are summarised in Table 7.

**Figure 13:**
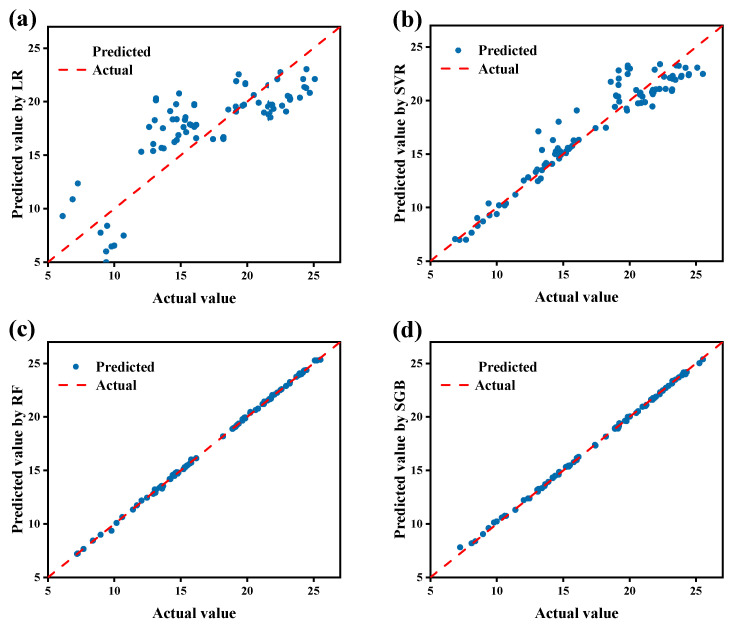
Performance comparison of actual versus predicted PCEs (%) for training and test datasets using (**a**) LR, (**b**) SVR, (**c**) RF, and (**d**) XGBoost models.

Model performance is quantified using the R^2^ and RMSE. The R^2^ value indicates the fraction of variation in the target variable captured by the model, with a value closer to 1 representing stronger agreement between the predicted and actual results, and generally, an R^2^ greater than 0.85 is considered indicative of good predictive capability. RMSE quantifies the typical deviation between predicted and actual values, with a smaller value indicating lower prediction error. Owing to its linear formulation, the LR model was unable to adequately describe the complex nonlinear dependencies within the dataset, resulting in comparatively poor predictive results. Although the R^2^ of the SVR model is close to the ideal value, its RMSE performance is poor, indicating that while the model could capture the overall trend of the data well, it suffered from shortcomings in local smoothness and accuracy in point-by-point prediction. Figure 13 compares the actual PCE curves with the predicted PCE curves obtained by all ML algorithms. The results indicate that RF and XGBoost provided the most accurate predictions among the evaluated models, with RF yielding the lowest RMSE. This excellent performance can be attributed to the structural characteristics of the dataset—the input parameters are continuous variables while the output is discrete. This configuration is empirically well-suited for tree-based ensemble methods [74]. Therefore, RF exhibited the best overall predictive performance among the models evaluated for this dataset.

**Figure 14:**
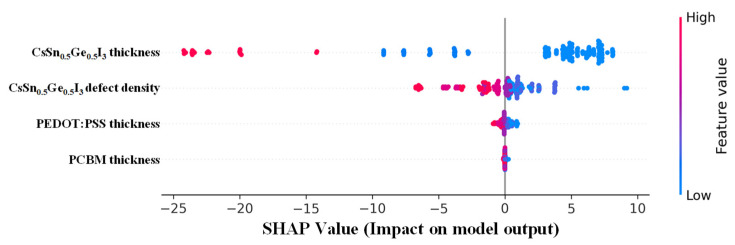
Feature importance for device PCE, derived from SHAP value analysis using a random forest model.

A quantitative analysis of feature importance based on the SHAP framework is performed on the Random Forest model, with the results presented in Figure 14. SHAP analysis indicates that the thickness of the CsSn_0.5_Ge_0.5_I_3_ absorber layer is one of the most critical parameters affecting PCE, with its reduction contributing significantly and positively to PCE improvement. In contrast, PEDOT:PSS and PCBM thicknesses show relatively minor contributions, while the defect density of CsSn_0.5_Ge_0.5_I_3_ has more pronounced effects on PCE. These findings can guide feature engineering strategies, such as focusing on the most influential parameters, removing variables with limited predictive value, and subsequently optimising the device structure.

#### 3.2.10. Optimised Device Properties

**Figure 15:**
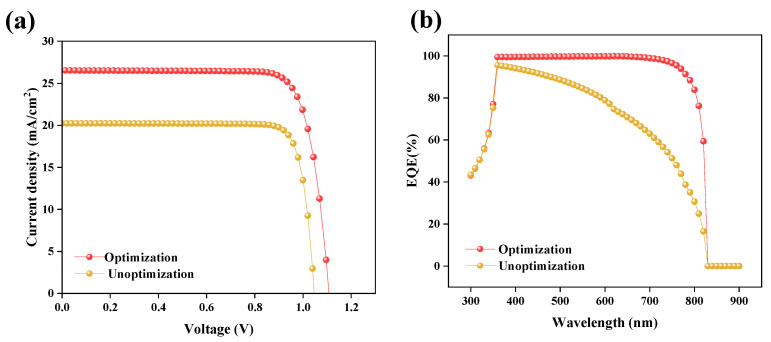
Comparison of (**a**) *J-V* characteristics and (**b**) EQE spectra.

Following optimisation, the device attained a PCE of 24.50%, a V_OC_ of 1.11 V, a J_SC_ of 26.52 mA·cm^−2^, and an FF of 80.01%. Figure 15a,b compare the *J-V* characteristics and external quantum efficiency (EQE) spectra of the device before and after optimisation, respectively. The optimised structure exhibited improvements in all evaluated photovoltaic parameters. The EQE began to increase at approximately 300 nm, remained at a relatively high level over the broad wavelength interval of 300–800 nm, and gradually decreased at longer wavelengths. These results suggest that the optimisation enhanced light harvesting and photogenerated-carrier collection across a wide spectral range. The optimised PCE of 24.50% should be regarded as a theoretical performance target obtained from an experimentally calibrated baseline model under substantially improved material and interface conditions, rather than as a currently demonstrated experimental efficiency. Experimentally approaching this performance will require further improvements in the absorber quality. The crystallinity and compositional uniformity of the absorber layer should be enhanced, while Sn^2+^ oxidation must be suppressed [75]. Bulk and interface defects should also be effectively passivated. In addition, energy-level alignment should be optimised [21], and resistive and non-radiative recombination losses should be minimised [76].

## 4. Conclusions

A collaborative strategy that integrates numerical simulation and machine learning is established for the modelling of all-inorganic CsSn_0.5_Ge_0.5_I_3_ PSCs. The optoelectronic properties of the CsSn_0.5_Ge_0.5_I_3_ absorber layer are investigated using first-principles calculations, and the structural configuration of the devices is optimised through SCAPS-1D simulations. Analysis of the energy-level alignment between the transport layers and the perovskite layer leads to the identification of FTO/PCBM/CsSn_0.5_Ge_0.5_I_3_/PEDOT:PSS/C as the optimal architecture, which exhibits superior photovoltaic performance. Systematic optimisation is performed for the thickness of the transport layers, the parameters of the absorber layer, and the back electrode configuration. Critical factors including resistance, illumination intensity, thermal stability, and carrier generation–recombination rates are examined in detail. The optimised device achieves a PCE of 24.50%, a V_oc_ of 1.11 V, a J_SC_ of 26.52 mA cm^−2^, and an FF of 80.01%. A systematic simulation and optimisation framework for improving the efficiency and stability of all-inorganic PSCs is thereby provided. To complement the numerical optimisation, four supervised ML algorithms, namely LR, SVR, RF, and XGBoost, are employed to predict device performance parameters. The RF model delivers the best predictive performance. Subsequent application of the SHAP framework to the random forest model quantifies the relative contribution of distinct structural features to energy-conversion efficiency. By combining numerical simulations with data-driven machine-learning techniques, this study establishes a reliable and interpretable strategy for developing high-efficiency and environmentally sustainable PSCs.

## Figures and Tables

**Table 1:** Simulation parameters.

Parameters	FTO	PCBM	CsSn_0.5_Ge_0.5_I_3_	Spiro-MeOTAD
Thickness (μm)	0.5	0.05	0.2	0.2
E_g_ (eV)	3.5	2	1.5	3
χ (eV)	4	3.9	3.9	2.2
$\varepsilon_{r}$	9	3.9	28	3
N_c_ (cm^−3^)	2.2 × 10^18^	2.5 × 10^21^	3.1 × 10^18^	2.2 × 10^18^
N_v_ (cm^−3^)	1.8 × 10^19^	2.5 × 10^21^	3.1 × 10^18^	1.8 × 10^19^
$\mu_{e}$ (cm^2^/(VS))	20	0.2	974	2.1 × 10^−3^
$\mu_{h}$ (cm^2^/(VS))	10	0.2	213	2.16 × 10^−3^
N_D_ (cm^−3^)	2 × 10^19^	2.93 × 10^17^	0	0
N_A_ (cm^−3^)	0	0	1 × 10^14^	1 × 10^18^
N_T_ (cm^−3^)	1 × 10^15^	1 × 10^15^	1 × 10^15^	1 × 10^15^
Reference	[36]	[37]	[38]	[37]

**Table 4:** Calculated electronic bandgap with available reported data.

	CsSnI_3_	CsSn_0.5_Ge_0.5_I_3_	CsGeI_3_
Calculated data	-	0.526	-
Reported data [51]	0.48	0.53	0.66

**Table 5:** CBO parameters across multiple ETL materials.

Material	Eg(eV)	χ (eV)	CBO
TiO_2_	3.2	4	−0.1
PCBM	2	3.9	0
ZnO	3.3	4	−0.1
IGZO	3.05	4.16	−0.26
WO_3_	3	4.16	−0.26

**Table 6:** VBO parameters across multiple HTL materials.

Material	Eg(eV)	χ (eV)	VBO
CuSCN	3.6	1.7	−0.1
P3HT	1.7	3.5	−0.2
PEDOT:PSS	1.6	3.4	−0.4
Spiro-MeOTAD	3	2.2	−0.2
CuI	3.1	2.1	−0.2

**Table 7:** Summary of various trained ML models.

ML Model	R^2^	RMSE
LR	0.7090	2.9269
SVR	0.9102	1.3331
RF	0.9999	0.0635
XGBoost	0.9993	0.1809

## Data Availability

The original contributions presented in this study are included in the article/Supplementary Materials. Further inquiries can be directed to the corresponding author.

## Supplementary Materials

The following supporting information can be downloaded at: https://www.mdpi.com/article/10.3390/ma19153341/s1, Table S1. Input parameters for interface defect layers; Table S2. Input parameters for the ETL materials; Table S3. Input parameters for the HTL materials; Table S4. Ionic radii used for calculating the tolerance factor and octahedral factor; Table S5. The dataset for training the ML model. References [77,78,79,80] are cited in the supplementary materials.
