# Supplementary material for: First-Principles DFT Investigation of CsSn0.5Ge0.5I3 and Machine Learning-Assisted Numerical Simulation of Lead-Free Solar Cells
Source: Materials (Basel). 2026 Aug 6;19(15):3341. doi: 10.3390/ma19153341 (PMC13468405; doi:10.3390/ma19153341)
Supplement: Supplementary file 1 [file materials-19-03341-s001.zip › materials-4451920-supplementary.pdf]

Supporting Information

First-Principles DFT Investigation of $CsSn_{0.5}Ge_{0.5}I_3$ and Machine Learning-Assisted Numerical Simulation of Lead-Free Solar Cells

Qinmiao Yu, Jinglan Liang, Xueji Chang, Xiaojuan Xia, Jiang Zhao**

College of Integrated Circuit Science and Engineering, Nanjing University of Posts and Telecommunications, Nanjing 210023, China

*Corresponding authors, E-mail: xiaxiaojuan@njupt.edu.cn (X.X.); jzhao@njupt.edu.cn (J.Z.)

1 SCAPS-1D optimization

SCAPS-1D software [1] is based on the drift-diffusion model, and is solved according to Poisson equation (S1), electron current density expression (S2), hole current density expression (S3), electron continuity equation (S4), hole continuity equation (S5). The steady state and transient electrical responses of perovskite solar cells (PSCs) under standard test conditions (AM1.5G spectrum, irradiance 1000 W/m^2, temperature 300 K) can be obtained by solving the partial differential equation simultaneously. The photoelectric conversion efficiency (PCE) is expressed in Eq. (S6).

$$\frac{d^2\psi(x)}{dx^2} = -\frac{q}{\varepsilon_0\varepsilon_r}\left[-n(x)+p(x)+N_D^+(x)-N_A^-(x)+\frac{\rho_{def}}{q}\right] \quad \text{(S1)}$$

$$J_n = n\mu_n\frac{d\psi}{dx}+D_n\frac{\partial n}{\partial x} \quad \text{(S2)}$$

$$J_p = p\mu_p\frac{d\psi}{dx}+D_p\frac{\partial p}{\partial x} \quad \text{(S3)}$$

$$-\frac{\partial J_n}{\partial x}-R_n+G=\frac{\partial n}{\partial t} \quad \text{(S4)}$$

$$-\frac{\partial J_p}{\partial x}-R_p+G=\frac{\partial p}{\partial t} \quad \text{(S5)}$$

$$\text{PCE}=\frac{P_{\text{max}}}{P_{\text{in}}}=\frac{J_{sc}\times V_{oc}\times \text{FF}}{P_{\text{in}}}\times 100\% \quad \text{(S6)}$$

In the above equation, ψ represents the electrostatic potential, ε_0 represents the dielectric constant of the vacuum, and ε_r represents the relative dielectric constant of the semiconductor. q represents the electron charge, where p represents the density of holes and n represents the equilibrium concentration of holes and electrons. N_D^+ refers to the equilibrium concentration of the ionizing donor, while N_A^- refers to the equilibrium concentration of the ionizing acceptor. The charge density is ρ, J_n represents the current density of the electron, and J_p represents the current density of the hole. R_n and R_p are the compound rate of electrons and holes, while G denotes

the formation rate. x denotes the position along the longitudinal axis of the device, and t denotes the time. In addition, D_n and D_p are the diffusivity of carriers, while μ_n and μ_p represent the mobility of electrons and holes.

2 Input Parameters

2.1 Input parameters in SCAPS-1D

Table S1. Input parameters for interface defect layers

Parameter	ETL/ $CsSn_{0.5}Ge_{0.5}I_3$	$CsSn_{0.5}Ge_{0.5}I_3$/ HTL
Defect type	Neutral	Neutral
σ_e (cm^{-2})	1×10^{-17}	1×10^{-18}
σ_h (cm^{-2})	1×10^{-18}	1×10^{-19}
E_r (eV)	0.6	0.6
Energy level state dispersion	Single	Single
Defect density (cm^{-3})	1×10^{10}	1×10^{10}
Reference	[2]	

Table S2. Input parameters for the ETL materials

Parameters	TiO_2	PCBM	ZnO	IGZO	WO_3
Thickness (μm)	0.03	0.05	0.05	0.03	0.05
E_g (eV)	3.2	2	3.3	3.05	3.12
χ (eV)	4	3.9	4	4.16	4.16
ε_r	9	3.9	9	10	10
N_c (cm^{-3})	2×10^{18}	2.5×10^{21}	3.7×10^{18}	5×10^{18}	5×10^{18}
N_v (cm^{-3})	1.8×10^{19}	2.5×10^{21}	1.8×10^{19}	5×10^{18}	5×10^{18}
μ_e ($cm^2/(VS)$)	20	0.2	100	15	15
μ_h ($cm^2/(VS)$)	10	0.2	25	0.1	0.1
N_D (cm^{-3})	9×10^{16}	2.93×10^{17}	1×10^{18}	1×10^{17}	1×10^{18}
N_A (cm^{-3})	0	0	0	0	0
N_T (cm^{-3})	1×10^{15}	1×10^{15}	1×10^{15}	1×10^{15}	1×10^{15}
Reference		[3]			[4]

Table S3. Input parameters for the HTL materials

Parameters	CuSCN	P3HT	PEDOT:PSS	Spiro-MeOTAD	CuI
Thickness (μm)	0.05	0.05	0.05	0.2	0.1
E_g (eV)	3.6	1.7	1.6	3	3.1
χ (eV)	1.7	3.5	3.4	2.2	2.1
ε_r	10	3	3	3	6.5
N_c (cm^{-3})	2.2×10^{19}	2×10^{21}	2.2×10^{18}	2.2×10^{18}	2.8×10^{19}
N_v (cm^{-3})	1.8×10^{18}	2×10^{21}	1.8×10^{19}	1.8×10^{19}	1×10^{19}
μ_e ($cm^2/(VS)$)	100	1.8×10^{-3}	4.5×10^{-2}	2.1×10^{-3}	100
μ_h ($cm^2/(VS)$)	25	1.86×10^{-2}	4.5×10^{-2}	2.16×10^{-3}	43.9
N_D (cm^{-3})	0	0	0	0	0
N_A (cm^{-3})	1×10^{18}	1×10^{18}	1×10^{18}	1×10^{18}	1×10^{18}
N_T (cm^{-3})	1×10^{15}	1×10^{15}	1×10^{15}	1×10^{15}	1×10^{15}
Reference			[3]		

2.2 Stability of $CsSn_{0.5}Ge_{0.5}I_3$ Materials

Table S4. Ionic radii used for calculating the tolerance factor and octahedral factor

Ion	Charge	Coordination	Ionic radii (Å)	Reference
Cs	+1	XII	1.88	[5]
Sn	+2	VI	1.02	[6]
Ge	+2	VI	0.73	[5]
I	-1	VI	2.20	[5]

2.3 Machine learning

Table S5. The dataset for training the ML model

Parameter	Range	Increment rate	Total number of steps
$CsSn_{0.5}Ge_{0.5}I_3$ thickness	0.1-1	0.225	5
$CsSn_{0.5}Ge_{0.5}I_3$ defect density	10^{12}-10^{18}	1(logarithmic)	7
PEDOT:PSS thickness	0.03-0.3	0.0675	5
PCBM thickness	0.03-0.3	0.0675	5

Reference

[1] M. Burgelman, P. Nollet, S. Degrave, Modelling polycrystalline semiconductor solar cells, Thin Solid Films 361–362 (2000) 527–532. https://doi.org/10.1016/S0040-6090(99)00825-1.

[2] M.K. Hossain, M.H.K. Rubel, G.F.I. Toki, I. Alam, Md.F. Rahman, H. Bencherif, Effect of Various Electron and Hole Transport Layers on the Performance of $CsPbI_3$-Based Perovskite Solar Cells: A Numerical Investigation in DFT, SCAPS-1D, and wxAMPS Frameworks, ACS Omega 7 (2022) 43210–43230. https://doi.org/10.1021/acsomega.2c05912.

[3] M.K. Hossain, G.F. Ishraque Toki, D.P. Samajdar, M.H.K. Rubel, M. Mushtaq, Md.R. Islam, Md.F. Rahman, S. Bhattarai, H. Bencherif, M.K.A. Mohammed, R. Pandey, J. Madan, Photovoltaic Performance Investigation of $Cs_3Bi_2I_9$-Based Perovskite Solar Cells with Various Charge Transport Channels Using DFT and SCAPS-1D Frameworks, Energy Fuels 37 (2023) 7380–7400. https://doi.org/10.1021/acs.energyfuels.3c00540.

[4] N. Rai, S. Rai, P.K. Singh, P. Lohia, D.K. Dwivedi, Analysis of various ETL materials for an efficient perovskite solar cell by numerical simulation, J Mater Sci: Mater Electron 31 (2020) 16269–16280. https://doi.org/10.1007/s10854-020-04175-z.

[5] R.D. Shannon, Revised effective ionic radii and systematic studies of interatomic distances in halides and chalcogenides, (n.d.). https://doi.org/10.1107/S0567739476001551.

[6] V. Sidey, On the effective ionic radii for the tin(II) cation, Journal of Physics and Chemistry of Solids 171 (2022) 110992. https://doi.org/10.1016/j.jpcs.2022.110992.
